# Prediction of Member Forces of Steel Tubes on the Basis of a Sensor System with the Use of AI

**DOI:** 10.3390/s25030919

**Published:** 2025-02-03

**Authors:** Haiyu Li, Heungjin Chung

**Affiliations:** Artificial Intelligence, Civil and Environmental Engineering, Jeonju University, Jeonju 55069, Republic of Korea; haiyuli@jj.ac.kr

**Keywords:** AI, offshore wind turbine support systems, steel tubes, FRP double-helix sensor system, SHM, ABAQUS, FCNN, CNN, GUI, member forces

## Abstract

The rapid development of AI (artificial intelligence), sensor technology, high-speed Internet, and cloud computing has demonstrated the potential of data-driven approaches in structural health monitoring (SHM) within the field of structural engineering. Algorithms based on machine learning (ML) models are capable of discerning intricate structural behavioral patterns from real-time data gathered by sensors, thereby offering solutions to engineering quandaries in structural mechanics and SHM. This study presents an innovative approach based on AI and a fiber-reinforced polymer (FRP) double-helix sensor system for the prediction of forces acting on steel tube members in offshore wind turbine support systems; this enables structural health monitoring of the support system. The steel tube as the transitional member and the FRP double helix-sensor system were initially modeled in three dimensions using ABAQUS finite element software. Subsequently, the data obtained from the finite element analysis (FEA) were inputted into a fully connected neural network (FCNN) model, with the objective of establishing a nonlinear mapping relationship between the inputs (strain) and the outputs (reaction force). In the FCNN model, the impact of the number of input variables on the model’s predictive performance is examined through cross-comparison of different combinations and positions of the six sets of input variables. And based on an evaluation of engineering costs and the number of strain sensors, a series of potential combinations of variables are identified for further optimization. Furthermore, the potential variable combinations were optimized using a convolutional neural network (CNN) model, resulting in optimal input variable combinations that achieved the accuracy level of more input variable combinations with fewer sensors. This not only improves the prediction performance of the model but also effectively controls the engineering cost. The model performance was evaluated using several metrics, including *R*^2^, *MSE*, *MAE*, and *SMAPE*. The results demonstrated that the CNN model exhibited notable advantages in terms of fitting accuracy and computational efficiency when confronted with a limited data set. To provide further support for practical applications, an interactive graphical user interface (GUI)-based sensor-coupled mechanical prediction system for steel tubes was developed. This system enables engineers to predict the member forces of steel tubes in real time, thereby enhancing the efficiency and accuracy of SHM for offshore wind turbine support systems.

## 1. Introduction

In the field of construction engineering, particularly in the structural design of offshore wind turbines, the support system is of significant importance. The steel tubes serve as transitional members, connecting the wind turbine and the foundation, which have the characteristics of fatigue capacity, buckling stability, and strong seismic performance, thus conferring substantial engineering benefits during construction [1,2,3,4]. In the case of offshore wind turbine (OWT) structures, which are mainly supported by monopiles, they are subjected to complex loads that change in real time, and real-time monitoring of the actual loads applied is required to predict the fatigue life of the structure and evaluate its soundness [5,6,7]. Strain gauges are essential sensors for measuring stress and displacement due to loads applied to structures and are widely used in strain-based SHM of structures. Sensors can be divided into discrete short-gauge sensors that measure strains in local areas and long-gauge sensors that can measure a wide range, such as fiber optic sensors. Short-gauge sensors provide measurements close to the exact (and not average) value of the strain. While this is important to understand local material behavior, it might lack the information at a global structural scale [8,9,10]. Therefore, to analyze the behavior of large structures such as OWTs, long–gauge sensors are required, and the quality of the analysis is determined by the location, spacing, and range of the sensors and the analysis method of sensor data.

SHM was first used in the early 2000s to monitor long-span bridges, and provides quantitative and reliable data on a structure’s integrity and detects degradations and cracks. The main challenges were maintaining these systems and analyzing the sensor data [11]. The advancement and implementation of ML algorithms in civil engineering have led to a growing application of AI-based machine learning models in structural engineering, particularly in the prediction of SHM and member forces, where AI has demonstrated promising performance [12,13,14,15]. The combination of extensive numerical data, growing computational power, and advanced AI algorithms represents a significant emerging technology for engineering structural design and force analysis studies [16,17,18,19,20].

In recent years, the application of Artificial Intelligence (AI) in Structural Health Monitoring (SHM) has made significant progress. Numerous studies have demonstrated the potential of AI methods in data generation, damage identification, and performance optimization. For instance, Luleci et al. utilized a 1-D deep convolutional generative adversarial network to generate vibration data related to structural damage, showcasing the application of AI in complex data generation [21]. Sabato et al. proposed an innovative SHM method for bridge health monitoring by combining non-contact sensing technologies with AI algorithms, focusing on image-based approaches [22].

In the field of deep learning, Azimi et al. provided a comprehensive review of DL methods in SHM, highlighting the role of sensor technology and UAV systems in supporting DL models [23]. Lagaros et al. further explored the latest developments in Artificial Neural Networks (ANNs) across multiple domains, emphasizing the importance of interdisciplinary collaboration [24]. These studies have provided theoretical and methodological references for the present research.

In terms of practical applications in SHM, several studies have closely integrated AI technologies with engineering practices. Chang et al. improved the accuracy of Tunnel Boring Machine (TBM) location prediction by combining finite element simulations with machine learning methods [25]. Zou et al. employed a Gaussian Process Model integrated with physical modeling for strain prediction in offshore wind turbine support structures, demonstrating its robustness under various operating conditions [26]. Similarly, Gualtero et al. achieved real-time parameter prediction for bridges by integrating multiple sensors with finite element models [27].

In summary, existing research demonstrates that AI technologies significantly enhance the monitoring and prediction of complex engineering structures. This study proposes a data-driven modeling algorithm that integrates AI technologies with the FRP double-helix sensor system, directly linking strain with reaction forces to predict the mechanical performance of steel tube members in offshore wind turbine support systems. The algorithm employs two deep learning models, FCNN and CNN. FCNN is capable of handling multi-input and multi-output nonlinear problems and is suitable for modeling the complex relationship between strain (input) and reaction forces (output). Its simple structure facilitates implementation and optimization, achieving high prediction accuracy with low computational costs on small-to-medium datasets. CNN, on the other hand, utilizes its local perception and parameter-sharing mechanisms to effectively capture spatial features in the data. This study innovatively transforms strain data into “image-like” representations and applies one-dimensional convolution operations to extract local correlations, improving both the generalization ability and computational efficiency of the model.

Furthermore, by combining finite element method (FEM)-generated simulation data with strain data from the FRP double-helix sensor system, this study employs FCNN and CNN models to achieve efficient prediction of strain–reaction force relationships. This method not only ensures predictive accuracy but also significantly reduces engineering costs and enhances the real-time capability of SHM. Such a data-driven ML approach provides new insights into data analysis and design in structural engineering, showcasing the vast potential of AI in civil engineering and laying a foundation for the expansion of future research directions.

## 2. ABAQUS Finite Element Analysis

### 2.1. Establishment of Finite Element Model

**Figure 1:**
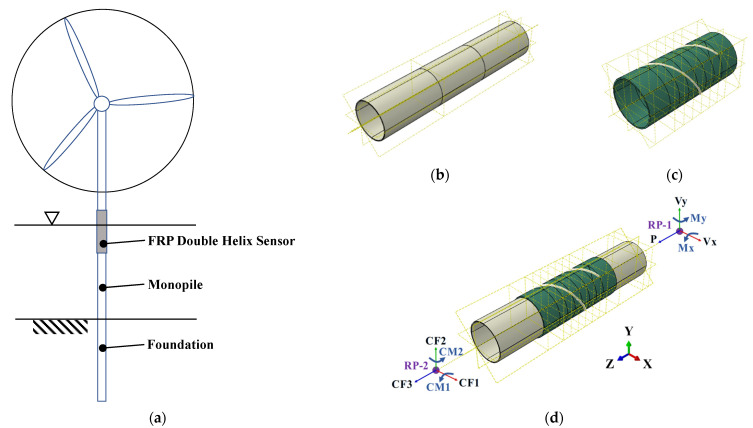
Numerical model of offshore wind turbine: (**a**) structural system; (**b**) monopile; (**c**) FRP double-helix sensor; (**d**) applied and reaction forces with orientation.

ABAQUS is a widely utilized FEA and computer-aided engineering (CAE) software for the assessment of engineering materials, mechanical components, and assemblies. The software boasts robust modeling and simulation capabilities, particularly in the simulation of intricate stress states and non-linear material behavior. ABAQUS is adept at precise 3D modeling and analysis of an array of engineering structures, offering intuitive visualization of the results [28,29,30]. In this experiment, ABAQUS/CAE (version number: 2022) was employed to model the transitional members (steel tube, FRP tube, and double-helix steel wire) in the offshore wind turbine support system with 3D solid FEM (as illustrated in Figure 1). To ensure the accuracy of the structural simulation results, all modeled materials are assumed to be homogeneous elastic materials with transversely isotropic material properties [31,32]. This material characteristic is more reflective of the performance differences between isotropic materials in different directions in actual engineering. The steel tube and FRP sensor are connected with bolts, and a contact element that considers friction is used between the two surfaces.

Furthermore, the specific physical properties of the relevant materials are listed in Table 1 to ensure consistency between the material properties in the model and the actual material parameters, thereby enhancing the reliability and validity of the analysis. Meanwhile, the FEM employed in this study offers a crucial theoretical foundation and practical assistance for the subsequent assessment of the mechanical properties and the optimized design of the transition members in the offshore wind turbine support system.

Two reference points, designated RP-2 and RP-1, are established on the axis of symmetry of the steel tube model. This is done in order to apply axial loads and bending moments at the aforementioned reference points, while simultaneously ensuring that the coupling constraints between the reference points and the top and bottom surfaces are met. Additionally, this approach allows for the realization of the desired loading conditions. The loads applied by RP-2 are in the negative direction of the *Z*-axis (CF3 = P’= −1000 N), the positive direction of the *X*-axis (CF1 = Vx’ = 50,000 N), and the positive direction of the *Y*-axis (CF2 = Vy’ = 50,000 N), and the applied bending moments are in the counterclockwise direction of the *X*-axis (CM1 = Mx’ = 3 × 10^6^ N·m). Furthermore, the loads are applied in a counterclockwise direction on the *Y*-axis (CM2 = My’ = 3 × 10^6^ N·m). It should be noted that RP-1 is fixed. The loads were applied in accordance with the amplitude curve (CF3: *axial_force*, CF1 and CM1: *sine*, CF2 and CM2: *sine2*), for a duration of one second, with a loading step of 1000.

**Figure 2:**
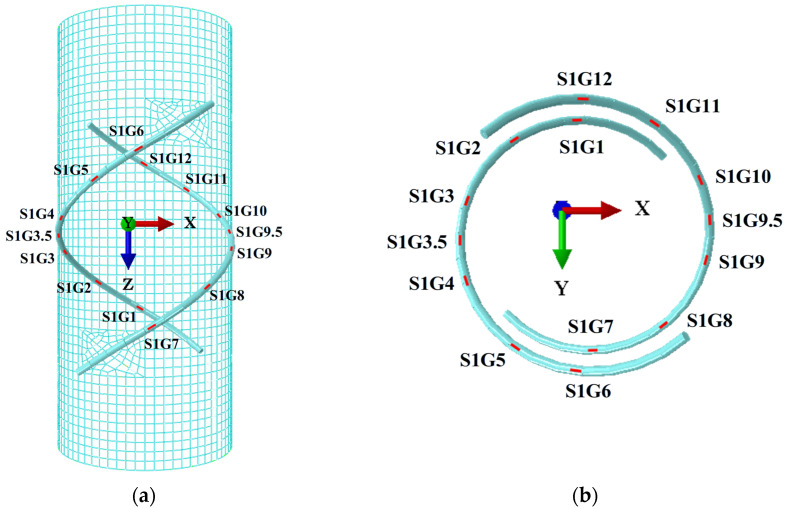
Strain distribution of the 14 cells of the FRP double-helix sensor: (**a**) XZ plane; (**b**) XY plane.

A composed sensor system is simulated using FRP tubes and double-helix steel wires. The double-helix steel wire is divided into 14 units (S1G1, S1G2, S1G3, S1G3.5, S1G4, S1G5, S1G6, S1G7, S1G8, S1G9, S1G9.5, S1G10, S1G11, S1G12), which are represented as 14 strain gauges affixed to the FRP tube. The measurement points are arranged symmetrically. When one end of the steel tube, RP-2, is subjected to force, the sensor system undergoes elastic deformation, and the 14 cells generate corresponding strain values. The other end, RP-1, generates corresponding reaction forces (P, Vx, Vy, Mx, My). The distribution of the 14 cells is illustrated in Figure 2.

### 2.2. Analysis of Numerical Simulation

**Figure 3:**
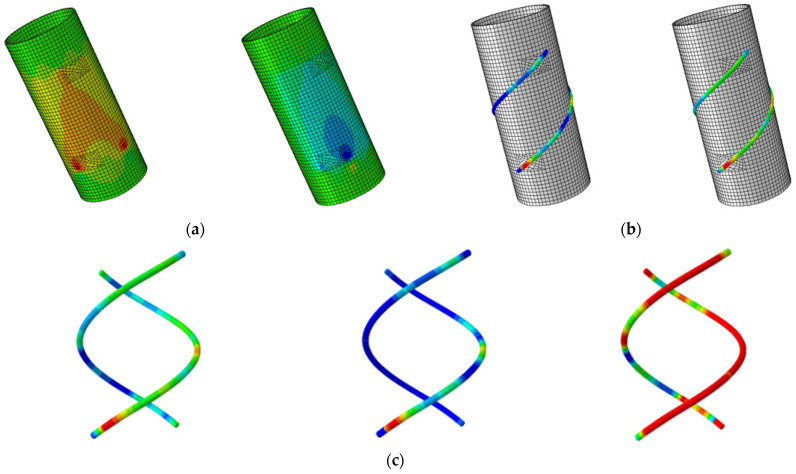
Typical results of FEM analysis: (**a**) equivalent stresses of FRP tube; (**b**) strain distribution of double-helix sensor; (**c**) strain patterns of fiber sensor wires.

The partial results of the ABAQUS FEA are shown in Figure 3. The figure illustrates the von Mises equivalent stress distribution of the FRP tube and the strain distribution along the length of the double-helix sensor. In the FEM, a refined mesh was applied to the local regions of the FRP tube and the double-helix sensor to accurately capture the stress and strain distributions. Additionally, the stress transfer relationship between the tube and the sensor was precisely modeled at the bolted connections. The strain results at 14 measurement points reveal the distribution pattern along the sensor’s length.

**Figure 4:**
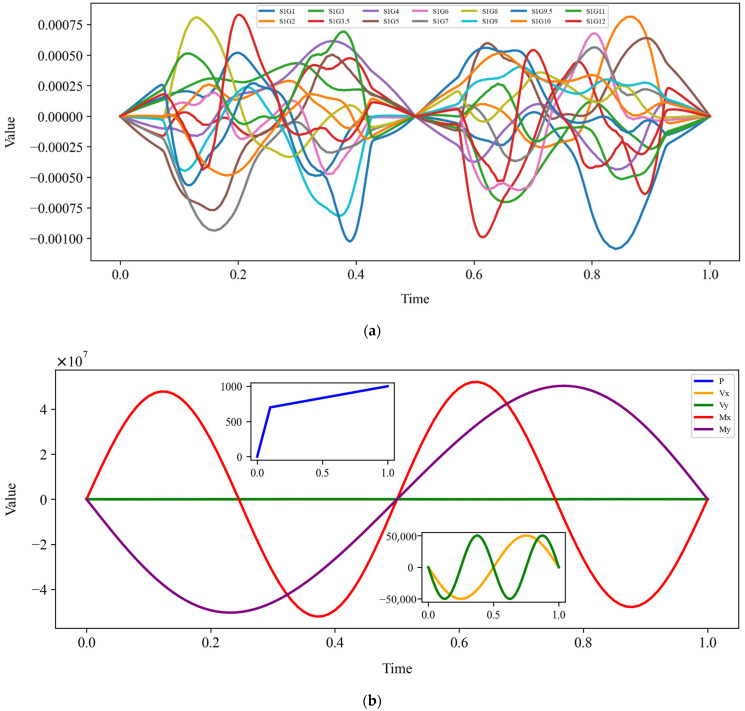
Plot of time vs. strain and branch reaction force: (**a**) time vs. 14 strains; (**b**) time vs. 5 reaction forces.

The ABAQUS field outputs the time for 1000 intervals with a duration of 1 s. Through numerical simulation, the relevant variables are output and the strain values of the 14 cells distributed on the FRP double-helix sensor are obtained, as are the reaction forces at the fixed end (RP-1). The data are presented in Table 2. The relationship between the FRP steel tubes and the strain and the reaction force under 1000 intervals of time of 1 s is illustrated in Figure 4. It can be observed that the time exhibits a nonlinear relationship with each strain, and that the time and the reaction force also become nonlinear under the loading of each amplitude. This nonlinear relationship also demonstrates the potential limitations of the model in addressing complex data patterns and extreme values. Consequently, the selection of the model should prioritize algorithms with nonlinear expressive capability. Furthermore, data preprocessing and regularization techniques should be emphasized during the training process to enhance the model’s generalization ability and robustness.

## 3. Introducing the Principles of Artificial Intelligence Machine Learning Models

In this paper, the study of multiple regression modeling for data interaction using DL techniques is explored in detail based on AI. The graphical user interface in the Anaconda navigator was used, with the help of the Jupyter Notebook platform (version no. 6.4.0) and Python programming language (version no. 3.8), and utilizing libraries such as NumPy (version no. 1.21.0) and Pandas (version no. 1.3.0), based on TensorFlow and the Feedforward Neural Network (FNN) of the Keras framework, to construct the FCNN model and CNN model [33,34,35,36]. The two models successfully simulated the nonlinear mapping relationship between 14 shape variables (S1G1–S1G12) and 5 force variables (P-My), and solved the multi-input and multi-output problems, which provided a reliable neural network modeling framework for the interaction between data science and ML, and then realized the accurate prediction of the coupled mechanical properties of ML models.

### 3.1. Data Analysis

According to the ABAQUS numerical simulation, to get the total 19 force variables of the steel tubes, using the time interval of 1000 for the field output, a number table containing 1000 rows and 19 columns (14 line strains as inputs and 5 reaction force variables as outputs) in Excel format was built. In this study, 80% (800) of the data points were used for the training set and the remaining 20% (200) of the data points were used as a test set of unknown data to test the accuracy and predictive performance of the training model, and the model was validated to have a certain degree of truthfulness and reliability. The data analysis was conducted using a variety of Python libraries to generate graphs and perform statistical calculations. These include Pandas for data processing, Matplotlib and Seaborn for visualization, NumPy and SciPy for numerical operations and statistical analysis, etc.

#### 3.1.1. Correlation Matrix Analysis

**Figure 5:**
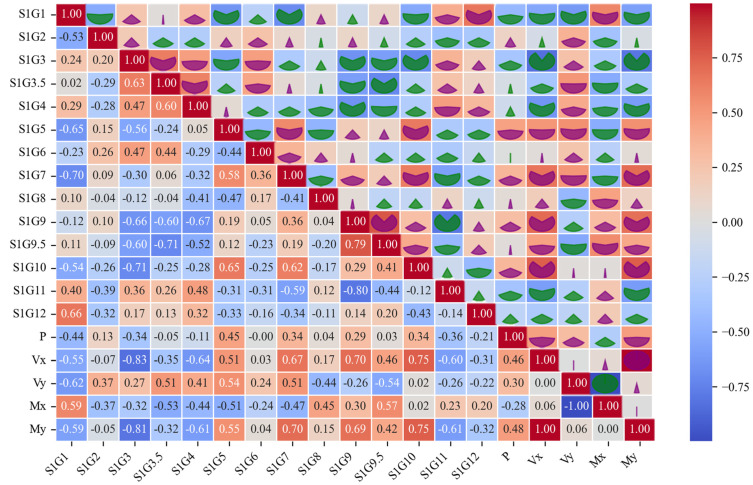
Heatmap of correlation matrix of input and output variables.

The relationships between the input and output variables are elucidated through the use of a correlation matrix heatmap (Heatmap), as illustrated in Figure 5. The values of the correlation coefficients are represented by shades of color, typically employing distinct color mappings [37,38,39]. For example, S1G1 exhibits a notable negative correlation with My, Vx, Vy, and P, while showing a positive correlation with Mx, making it an important input variable. S1G7 is a key variable, displaying a strong positive correlation with My and a significant negative correlation with Mx. S1G9.5 has a significant negative correlation with Vy, making it a critical variable for representing Vy in the model.

#### 3.1.2. Statistical Analysis of Variables

The numerical statistical characterization of the input and output variables is illustrated in Table 3 [40,41]. The numerical ranges of the input strains are small, with the maximum and minimum values fluctuating on the order of 10^−4^, with the mean close to zero and a small standard deviation, reflecting a small range of fluctuation; in contrast, the numerical ranges of the output reaction force variables are significantly larger, with the maximum and minimum values on the order of 10^3^ to 10^7^, respectively, especially for Mx and My, which exhibit a high degree of dispersion in their data distributions. The analysis of skewness and kurtosis revealed that some of the variables (e.g., S1G8, S1G2, etc.) were characterized by skewed distributions, with most of the kurtosis values being low and close to the normal distribution, whereas the kurtoses of P and Vy were high and the skewness was low, displaying a slight negative skewness, suggesting that the distributions of these variables had a tendency to be concentrated. Overall, the statistical analysis provides the basis for subsequent model construction and variable screening, revealing significant differences in the values and distributional characteristics of the input and output variables.

**Figure 6:**
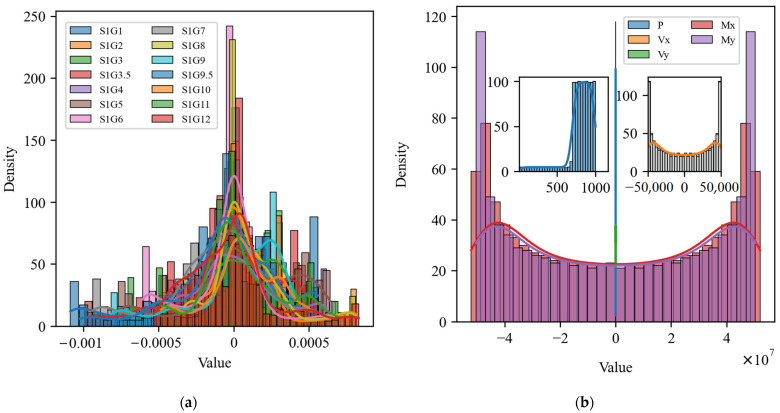
Histograms and probability density plots of the distribution of input and output variables: (**a**) 14 input variables; (**b**) 5 output variables.

Furthermore, Figure 6 depicts the histograms and probability density plots of the input and output variables [42,43,44]. The distributions of the majority of input variables in subfigure (a) exhibit pronounced bimodal characteristics and substantial fluctuations around 0. The data exhibit bimodal characteristics, with kurtosis concentrated around 0. In contrast, subfigure (b) illustrates that the distribution of output variables is symmetrical, particularly in the Vx, Vy, Mx, and My variables, which display the characteristics of a U-shaped distribution, while the distribution of P variables is more concentrated. Overall, the distributional characteristics of the input and output variables demonstrate disparate numerical properties, with the input variables exhibiting a more intricate pattern and the output variables displaying a more concentrated distribution. This discrepancy in distribution patterns suggests a potential correlation between the data sets pertaining to the input strains and the output reaction force variables.

In conclusion, this study employed correlation matrix analysis, statistical eigenanalysis, and histogram and probability density plot analysis of input and output variables to gain a deeper understanding of the data characteristics, with the objective of optimizing the construction of the subsequent model. Correlation matrix analysis reveals the linear relationships among input variables, thereby facilitating the identification of potential multicollinearity issues and, consequently, guiding the variable screening and data processing. The analysis of statistical features provides information on the concentration trend, dispersion, skewness, and kurtosis of the variables. This analysis enables the determination of whether the data require standardization or normalization in order for the model to remain stable across different feature scales. In contrast, histograms and probability density plots offer a visual representation of the data distribution, allowing for the identification of skewness, outliers, and non-normal properties. This provides a foundation for selecting an appropriate model, such as a linear model or a deep learning model. These data analysis steps not only enhance the understanding of the data structure, but also provide critical support for the validity and generalizability of subsequent models.

### 3.2. FCNN Model Construction and Analysis Study

The FCNN model comprises an input layer, one or more hidden layers, and an output layer, and the entire model exhibits a high degree of nonlinear expressiveness. The input layer represents the initial stage of the neural network, tasked with accepting input values and transmitting them to the subsequent layer. In contrast, the hidden layer comprises numerous neurons and is fully connected, executing diverse operations on the input data with corresponding weights and biases [45,46,47,48].

**Figure 7:**
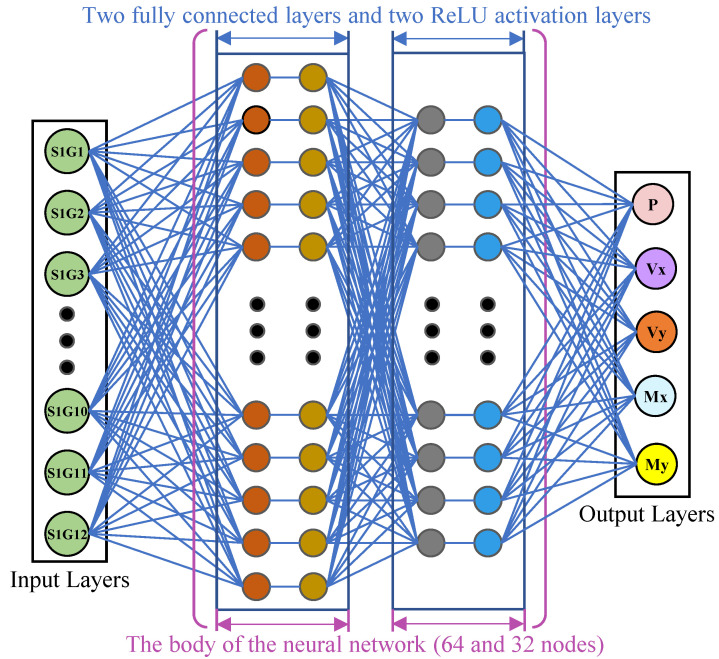
Schematic diagram of FCNN multiple regression model.

The FCNN model presented in this paper comprises an input layer, two hidden layers, and an output layer. The input layer is responsible for accepting the input data, the first hidden layer contains 64 nodes, and the second hidden layer contains 32 nodes. The final layer of the network is responsible for the output, which is represented by five variables: P, Vx, Vy, Mx, and My. The FCNN model was trained for 100 training cycles with a batch size of 32, and the learning rate was set to 0.01. The schematic diagram of the AI neural network multiple regression modeling engine is presented in Figure 7.

The core principles of the specific build are as follows.

- Step 1: Data preprocessing.

1. The data in the input layer were scaled using the maximum absolute value of each feature, with the objective of placing the values within the range of (−1, 1) [49,50].

(1) $$x^{\prime }=\frac{x}{max(|x|)}$$

In the formula, *max*(|*x*|) represents the maximum value of the absolute value of the sample data for the feature. The value of each feature in the sample data is represented by *x*, while *x’* denotes the value after normalization.

- Step 2: Construct a FCNN model. The formulas are represented as follows [51,52].

(2) $$Z^{\left(\mathrm{out}\right)}=Z^{\left(n+1\right)}=\sigma^{\left(n\right)}(\cdots \sigma^{\left(2\right)}(W^{{\left(2\right)}^{T}}\times (\sigma^{\left(1\right)}(W^{{\left(1\right)}^{T}}\times Z^{\left(0\right)}+B^{\left(1\right)})+B^{\left(2\right)})\cdots +B^{\left(n\right)})$$

1. *Z*^(0)^ represents the input data, which is expanded into the matrix form represented below [53].

(3) $$Z^{\left(0\right)}=\left[\begin{array}{c} z_{1},z_{2},...,z_{j},...,z_{n} \end{array}\right]$$

2. $W^{{\left(1\right)}^{T}}$ represents the weight matrix utilized in the linear transformation of the initial layer of the network. This matrix is expanded into the form represented below [54].

(4) $$W^{{\left(1\right)}^{T}}={\left[\begin{array}{cccccc} w_{11} & w_{12} & \cdots & w_{1j} & \cdots & w_{1n_{0}}\\ w_{21} & w_{22} & \cdots & w_{2j} & \cdots & w_{2n_{0}}\\ \vdots & \vdots & \ddots & \vdots & \ddots & \vdots \\ w_{j1} & w_{j2} & \cdots & w_{jj} & \cdots & w_{jn_{0}}\\ w_{n1} & w_{n2} & \cdots & w_{nj} & \cdots & w_{nn_{0}} \end{array}\right]}^{T}$$

3. *B*^(1)^ represents the bias matrix in the linear transformation of the initial layer of the network. This matrix is expanded into the form represented below [55].

(5) $$B^{\left(1\right)}={\left[b_{1},b_{2},...,b_{j},...,b_{n}\right]}^{T}$$

4. *σ*^(1)^ represents the nonlinear transformation in the initial layer of the network, expressed through the activation function rectified linear unit (ReLU). The formulas are expressed as follows [56,57,58,59].

(6) f(x)=max(0,x)

- Step 3: Construct the loss function to perform forward propagation. In this paper, the total loss function is equal to the weighted sum of five sub-loss functions, as expressed by the following formula [60,61].

(7) $$Loss_{total}=w_{P}\times {\mathrm{Loss}}_{P}+w_{V_{x}}\times {\mathrm{Loss}}_{V_{x}}+w_{V_{y}}\times {\mathrm{Loss}}_{V_{y}}+w_{M_{x}}\times {\mathrm{Loss}}_{M_{x}}+w_{M_{y}}\times {\mathrm{Loss}}_{M_{y}}$$

1. The assessment of each sub-loss within the model was conducted utilizing the *MSE’* metric [62,63].

(8) $$MSE^{\prime }=\frac{1}{n}\overset{n}{\underset{i=1}{\sum }}{\left(Y_{i}-{\hat{Y}}_{i}\right)}^{2}$$

- Step 4: Backpropagation is performed using gradient descent and the adaptive moment estimation (Adam) optimizer. By adaptively adjusting the learning rate of each parameter and updating all weight values, including those of the five sub-loss functions, the parameters are updated in a more independent manner. Concurrently, the dynamic adjustment of the parameters is employed to enhance the performance of the model and to generate the final predicted value that is closer to the actual value of the force of support and reaction. The formula for updating the weights is expressed as follows [64,65,66,67].

(9) $$w_{i,j}^{{\left(l\right)}^{\prime }}=w_{i,j}^{\left(l\right)}-\rho \frac{\partial Loss(W)}{\partial w_{i,j}^{\left(l\right)}}$$

1. Among the aforementioned formulas, that which expresses the chain rule for the calculation of the gradient is as follows [68].

(10) $$\frac{\partial Loss(W)}{\partial w_{i,j}^{\left(l\right)}}=\frac{\partial y_{j}^{\left(l\right)}}{\partial w_{i,j}^{\left(l\right)}}\times \frac{\partial z_{j}^{\left(l+1\right)}}{\partial y_{j}^{\left(l\right)}}\times \frac{\partial y^{\left(l+1\right)}}{\partial z_{j}^{\left(l+1\right)}}\times \frac{\partial z^{\left(l+2\right)}}{\partial y^{\left(l+1\right)}}\times \frac{\partial y^{\left(l+3\right)}}{\partial z^{\left(l+2\right)}}\times \cdots \times \frac{\partial y^{\left(L\right)}}{\partial z^{\left(L\right)}}\times \frac{\partial z^{\left(L+1\right)}}{\partial y^{\left(L\right)}}\times \frac{\partial Loss(W)}{\partial z^{\left(L+1\right)}}$$

### 3.3. CNN Model Construction and Analysis Study

CNN is a machine learning method that has been developed for the processing of image data. Its functionality is based on the combination of specific structures, including a convolutional layer (Conv1D), a pooling layer (MaxPooling1D) and a fully connected layer. These structures enable the extraction and classification of image features [69,70,71]. In contrast, the CNN model presented in this paper treats the input table of 1000 rows and *n* columns of numbers as an image format (where *n* represents the total input feature variable), representing a distinctive approach to data processing. This method maps the strain to the support reaction force, establishing a corresponding relationship. Consequently, the CNN model is capable of capturing a greater degree of positional information during the processing of data, which ultimately results in enhanced accuracy and generalization capabilities when addressing image-related problems.

Meanwhile, CNN models effectively capture positional information within input data through features such as sparse connectivity, parameter sharing, local perception, spatial invariance, and pooling operations. Sparse connectivity links convolutional kernels to localized regions, reducing the number of parameters and focusing on local features. Parameter sharing significantly decreases computational costs and enhances the stability of feature extraction. Local perception enables the model to capture relationships between adjacent data points, while spatial invariance extracts features that are robust to translations or rotations through convolution and pooling operations. Pooling further compresses data dimensions while retaining critical features.

**Figure 8:**
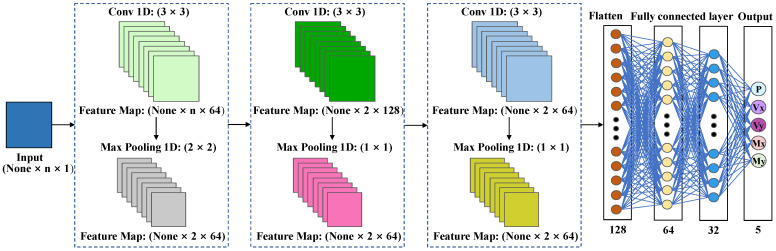
Schematic diagram of CNN multiple regression model.

The combination of these functionalities allows CNNs to excel in image and vision tasks by effectively capturing complex spatial relationships and improving generalization capabilities. In this study, CNNs process strain data in an “image-like format”, capturing the nonlinear mapping between strain and support reactions, thereby significantly enhancing prediction performance and optimizing computational efficiency [72]. The detailed construction process of the CNN model is illustrated in Figure 8.

The core principles of the specific build are as follows.

- Step 1: Data preprocessing. The calculations were performed in accordance with the requirements of Formula (1).
- Step 2: Define the primary structural framework of the CNN. The CNN model presented in this paper comprises three convolutional layers and the corresponding three maximum pooling layers [73].

1. Calculate the net input *Z^p^* of Conv1D. The convolution kernel (*W^p^*^,1^, *W^p^*^,2^, ⋯, *W^p,^^D^*) is used to convolve the input characteristic mappings (*X*^1^, *X*^2^, ⋯, *X^D^*), then the convolution results are summed and a scalar bias *b^p^* is added to finally obtain the net input *Z^p^* of Conv1D [74]. The formulas are represented as follows.

(11) $$Z^{p}=W^{p}⊗X+b^{p}=\overset{D}{\underset{d=1}{\sum }}W^{p,d}⊗X^{d}+b^{p}$$

2. Compute the output characteristic mapping *Y^p^* of Conv1D. Subsequently, the net input *Z^p^* of the Conv1D is passed through the ReLU nonlinear activation function, thereby obtaining the output feature mapping *Y^p^*. The weights of the convolution kernel and the bias are updated iteratively through the computation of the fully connected layer [75]. The ReLU activation function is employed for each Conv1D, with inputs and outputs consistently populated to ensure compatibility in size. The ReLU activation function was calculated in accordance with the requirements of Formula (6).
3. Calculate MaxPooling1D. The MaxPooling1D function is designed to extract the maximum value within each window, thereby reducing the spatial dimension of the input data set. This approach has the additional benefits of reducing the amount of computation and the number of parameters, while maintaining the integrity of the data’s salient features [76,77].

(12) $$MaxPooling1D\left(Y\right)=max\left(Y\right)$$

In the aforementioned formula, *Y* represents the tensor of the input MaxPooling1D, while the *max(Y)* function denotes the maximum value obtained within the *Y* tensor.

- Step 3: Flatten the layer. It is essential to flatten the output of the MaxPooling1D operation in order to establish a connection with the subsequent fully connected layer [78].
- Step 4: The fully connected layer. The fully connected layer neural network comprises two hidden layers, with 64 and 32 nodes, respectively, which are activated through the application of the ReLU function. The output layer is constituted by five nodes, which are responsible for predicting five values. The fully connected layer and the output layer are calculated in accordance with the FCNN principle [79].

### 3.4. Comparative Analysis of the FCNN Model and the CNN Model

The comparative advantages and disadvantages of the FCNN and CNN models are presented in Table 4. In summary, the advantages of the CNN model can be attributed to its capacity to extract and process spatial information. Furthermore, it is well suited for processing large datasets, as it can reduce computational complexity through parameter sharing. This makes it an excellent choice for tasks involving structured features, such as images and videos. In contrast, FCNN is more suited to addressing conventional machine learning tasks, such as classification and regression problems involving tabular data. It demonstrates superior performance in scenarios involving smaller datasets. However, it is susceptible to issues such as overfitting, which can be mitigated through techniques like regularization [80].

## 4. Construction of Analytical Indicators for Model Evaluation

### 4.1. Error Indicators

In this study, the coefficient of determination (*R*^2^), mean square error (*MSE*), mean absolute error (*MAE*), and symmetric and relative mean absolute percentage error (*SMAPE*) are selected as the overall evaluation indexes of the model to assess and analyze the prediction results of the model [81,82,83,84]. The *R*^2^ coefficient reflects the degree of model fitting, with a value closer to 1 indicating a superior fit. The *MSE* and *MAE* coefficients indicate the deviation between the predicted and actual values. A smaller deviation indicates greater accuracy. The *SMAPE* coefficient is a percentage-based error indicator. A smaller range of relative difference between the actual and predicted values indicates a superior prediction performance. The specific formula is as follows:

(13) $$R^{2}=1-\frac{\sum_{i=1}^{n}{\left(y_{i}-{\hat{y}}_{i}\right)}^{2}}{\sum_{i=1}^{n}{\left(y_{i}-\bar{y}\right)}^{2}}$$

(14) $$MSE=\frac{1}{n}\sum_{i=1}^{n}{\left(y_{i}-{\hat{y}}_{i}\right)}^{2}$$

(15) $$MAE=\frac{1}{n}\sum_{i=1}^{n}\left|y_{i}-{\hat{y}}_{i}\right|$$

(16) $$SMAPE=\frac{1}{n}\overset{n}{\underset{i=1}{\sum }}\frac{100\left|y_{i}-{\hat{y}}_{i}\right|}{(\left|y_{i}\right|+\left|{\hat{y}}_{i}\right|)/2}\%$$

In the formula, *n* represents the number of data points, *y_i_* denotes the actual value of the *i*th data point, *ŷ_i_* denotes the predicted value of the *i*th data point, and $\bar{y}$ denotes the average of the actual values.

### 4.2. Linear Correlation Strength Indicator

The strength of the linear correlation between the experimental and predicted values of the model’s training and test sets is represented by the Pearson correlation coefficient (PCC), along with a scatterplot to demonstrate the nonlinear expressiveness and model robustness. The calculation formula is provided below [85,86].

(17) $$R=\frac{\overset{n}{\underset{i=1}{\sum }}\left(x_{i}-\bar{x}\right)\left(y_{i}-\bar{y}\right)}{\sqrt{\overset{n}{\underset{i=1}{\sum }}{\left(x_{i}-\bar{x}\right)}^{2}\overset{n}{\underset{i=1}{\sum }}{\left(y_{i}-\bar{y}\right)}^{2}}}$$

In the formula, *R* represents the maximum correlation coefficient. A value that is closer to 1 indicates a superior model fit. *n* denotes the number of data points. The *x_i_* and *y_i_* values represent the *i*th observations of the two variables, respectively. The $\bar{x}$ and $\bar{y}$ values represent the mean values of the two variables, respectively.

## 5. Analysis of Model Predictions

### 5.1. FCNN Model for Cross-Comparison Prediction Analysis of Input Variables

In this study, six cross-combinations of 14 input variables are employed as inputs to the FCNN model, specifically Input 2, Input 4, Input 6, Input 10a, Input 10b, and Input 14. To ensure the reliability of the measurement results, the measurement points are arranged in a symmetrical fashion and added in a symmetrical manner at different locations. The symmetrical arrangement serves to eliminate systematic deviations that may be caused by the uneven distribution of measurement points, thereby equalizing the data coverage and measurement accuracy. This, in turn, improves the stability and representativeness of the data. Moreover, a comprehensive comparative analysis is conducted following the arrangement of each set of measurement points. This analysis assesses the impact of different arrangement schemes on the predictive accuracy of the model, based on the predictive performance of the model. This approach guarantees the dependability of the prediction outcomes obtained under diverse measurement point configurations, furnishing more valuable data for the subsequent optimization of the model.

#### 5.1.1. Comparative Analysis of Total Loss Function Curves

**Figure 9:**
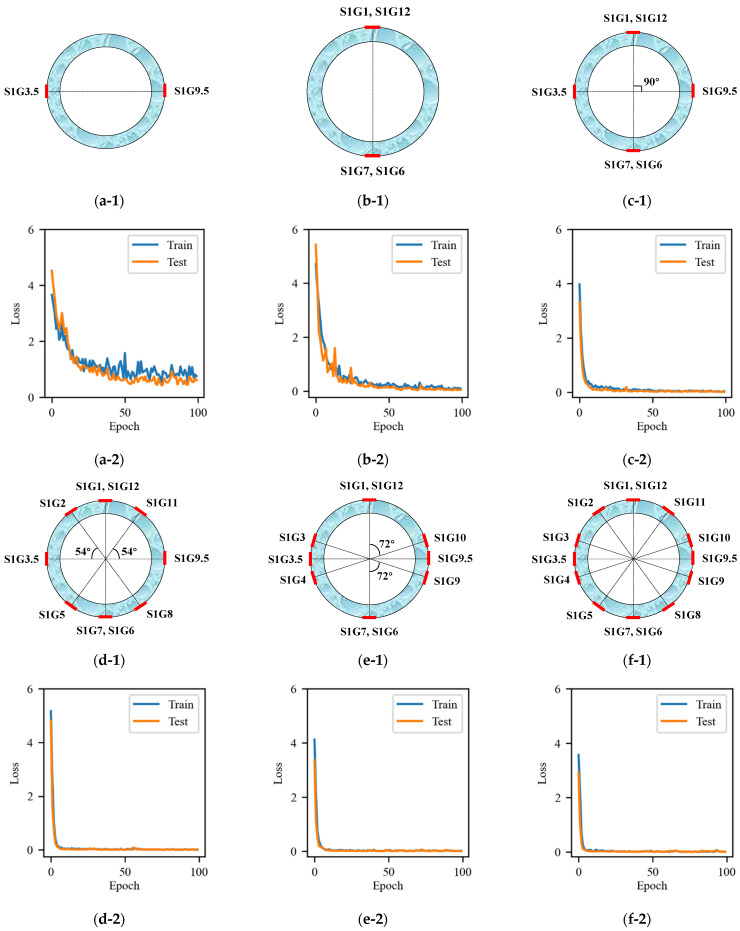
Strain combination and total loss function curves for FCNN model inputs: (**a**) Input 2; (**b**) Input 4; (**c**) Input 6; (**d**) Input 10a; (**e**) Input 10b; (**f**) Input 14.

The total loss function curves for each combination of six different input variables in the case of cross-combination are illustrated in Figure 9. Experimental results indicate that the number of input variables plays a critical role in model performance, directly affecting the model’s ability to capture data complexity. Increasing the number of input variables generally enhances the model’s ability to analyze complex data patterns, but it may also introduce redundant information, reducing the model’s generalization capability. Conversely, reducing input variables can lead to difficulty in capturing key features, resulting in a decline in prediction performance.

This study systematically investigated the impact of variable quantity on model performance by comparing different combinations (e.g., Input 2, Input 6, and Input 10b). The results showed that Input 2, due to its limited number of variables, failed to capture complex nonlinear features, leading to significantly lower prediction accuracy compared to other combinations. In contrast, the full-variable combination (Input 14) demonstrated the best performance in terms of accuracy and fitting ability, as well as the least variability, though it posed potential risks of overfitting. Ultimately, selecting a moderate number of variables (Input 10b) achieved a balance between performance and efficiency. This finding underscores that optimizing variable combinations not only reduces redundancy but also effectively controls model complexity, thereby improving overall model performance and adaptability.

The loss function curves of the FCNN model exhibit significant differences under different input combinations. The combination of a small number of variables (e.g., Input 2 and Input 4) results in a decrease in loss value at the outset of the training period. However, this combination also exhibits higher volatility and a higher final loss value in the middle and late periods. This indicates that the model’s ability to capture the complex features of the data is limited when a small number of variables are used, resulting in a reduction in prediction accuracy. Although the addition of Input 4 resulted in a slight improvement in model performance compared to Input 2, it still failed to meet the requisite accuracy for prediction, indicating that the model has a high demand for the number of input variables. The introduction of additional combinations of variables (e.g., Input 6) resulted in an improved descending speed and final stability of the loss curve, as well as a significantly lower final converged loss value than that observed for Input 4. This suggests that an increase in the number of input variables enhances the model’s ability to capture the complexity of the data, thereby improving prediction accuracy. This approach strikes a better balance between smoothness and accuracy, demonstrating reasonable performance. In particular, the loss curves of the Input 10a and Input 10b combinations exhibit fast convergence and low volatility, thereby further enhancing the stability and generalization performance of the model while improving its ability to capture non-linear features. In contrast, although the all-variable combination (Input 14) has the lowest loss value, the best accuracy, and low volatility, its computational cost and overfitting risk are relatively high. While this combination is suitable for application scenarios requiring high accuracy, it may be necessary to balance the computational cost in practical engineering applications.

#### 5.1.2. Analysis of Evaluation Results

- Comparative Analysis of Error Indicators.

**Figure 10:**
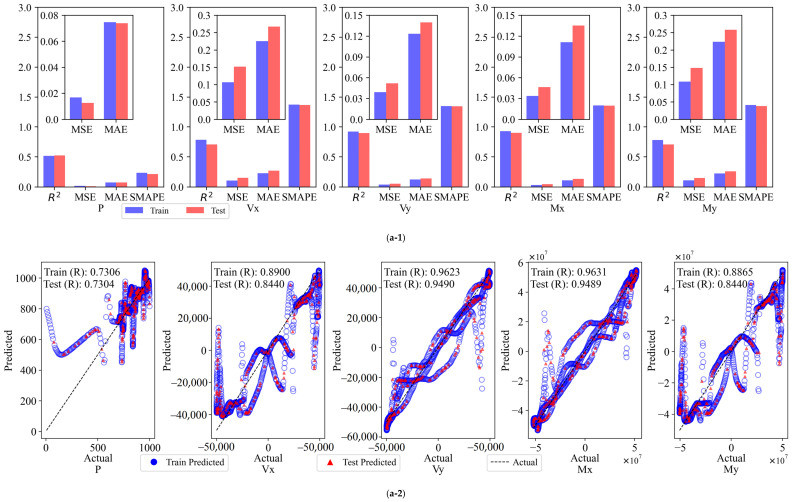

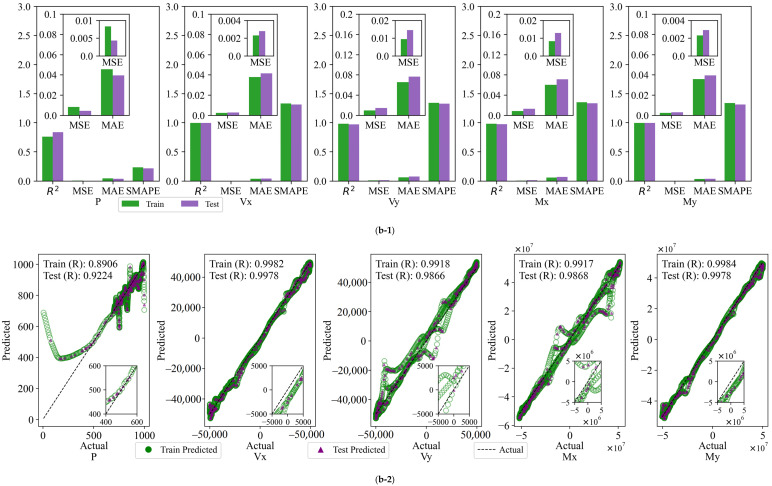

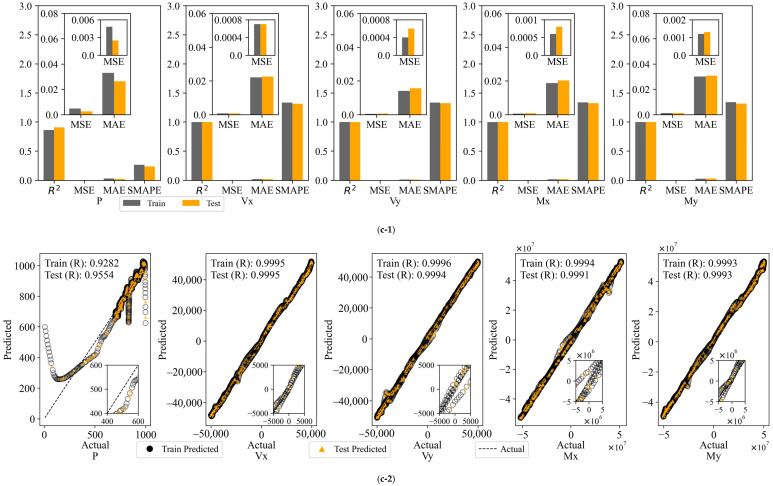

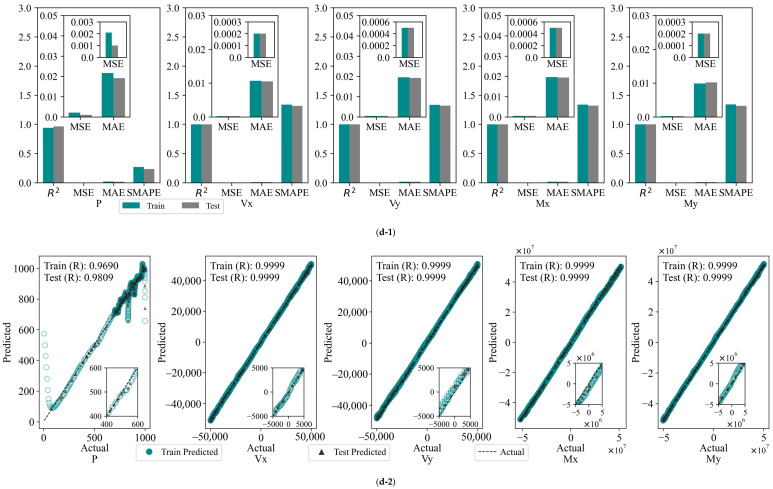

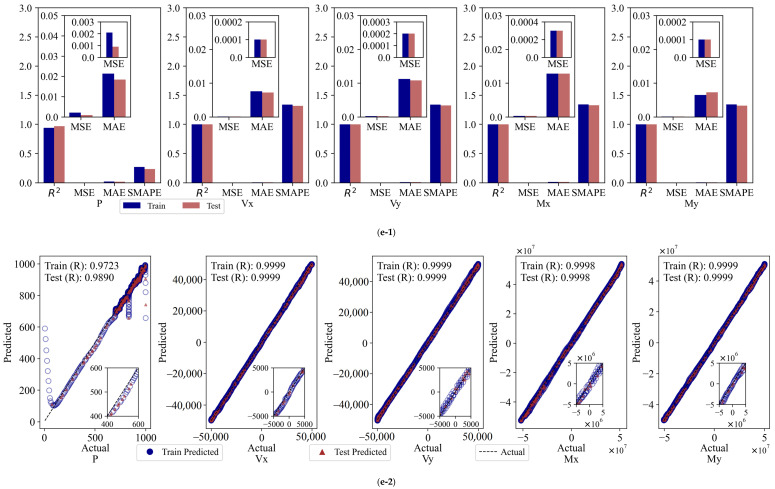

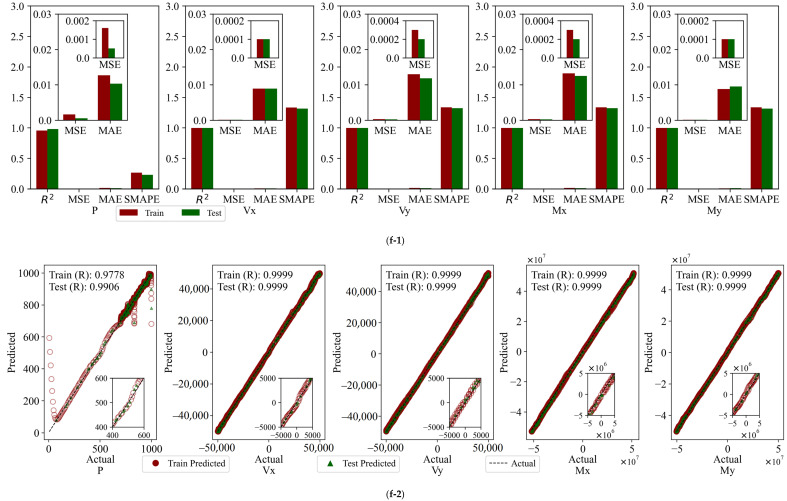
Evaluation metrics and linear correlation strength for FCNN models: (**a**) Input 2; (**b**) Input 4; (**c**) Input 6; (**d**) Input 10a; (**e**) Input 10b; (**f**) Input 14.

The FCNN model was employed to calculate the results of the assessment indexes for the six cases, which were then compared and subjected to statistical analysis. The model provides the values of *R*^2^, *MSE*, *MAE*, and *SMAPE* for the six cases within the same dataset, for both the training and test sets. These values are illustrated in Figure 10(a-1,b-1,c-1,d-1,e-1,f-1).

The *R*^2^ value of the model for the Input 2 combination is low, and the *MSE* and *MAE* are high, particularly in the prediction of outputs P and My. This indicates that a small number of input variables cannot adequately capture the complex features in the data. The introduction of additional variables in Input 4 resulted in an *R*^2^ value close to 1 for Vx and My, accompanied by a notable reduction in the *MSE* and *MAE*. However, the prediction of Vx and Vy remains subject to certain volatility. The Input 6 combination further enhances the model fit, with *R*^2^ values exceeding 0.9062 on both the training and test sets, and *MSE* and *MAE* markedly reduced, indicating that augmenting the number of variables facilitates the model’s ability to discern nonlinear relationships in the data. Of the 10-variable combinations, both Input 10a and Input 10b demonstrate optimal prediction accuracy and stability, with *R*^2^ values approaching 1 on both the training and test sets, lower *MSE* and *MAE*, and *SMAPE* remaining stable. This indicates that these two combinations not only effectively capture nonlinear features but also exhibit robust generalization performance, which is well suited for practical application requirements. The full-variable combination (Input 14) demonstrates the optimal performance, with an *R*^2^ value approaching 1, the lowest *MSE* and *MAE*, and a *SMAPE* within the minimum range on both the training and test sets. This indicates that the model is capable of comprehensive and precise fitting and prediction of each output variable.

- Comparative Analysis of the Strength of Linear Correlation.

Following the backscaling of the data, the linear correlation strength relationship curves between the predicted and true values of the FCNN model training and test sets are presented in Figure 10(a-2,b-2,c-2,d-2,e-2,f-2).

As the number of input variables increases, the linear correlation strength *R* between the predicted and experimental values of the FCNN model gradually improves, and the aggregation of data points around the diagonal is significantly enhanced, indicating enhanced predictive consistency. With a limited number of variable combinations (e.g., Input 2 and Input 4), the data points exhibit less aggregation around the diagonal and greater dispersion. Notably, the predictions of outputs P and My deviate significantly, with weaker linear correlation. Upon increasing the number of variables to six (Input 6), a notable tightening of the data points was observed, particularly in the prediction of Vx, Vy, and Mx. This resulted in a significant enhancement of the model fit, accompanied by a substantial increase in the *R*-value. For the “10”-variable combinations (Input 10a and Input 10b), the data points are distributed in a relatively uniform manner along the diagonal, and the *R*-value approaches 1, indicating a stronger linear correlation. For the full-variable combination (Input 14), the data points are observed to fit almost entirely on the diagonal, exhibiting a high degree of clustering. The resulting *R*-value is close to 1, indicating optimal agreement between the predicted and experimental values.

- Comparative Analysis of Comprehensive Evaluation Indicators.

**Figure 11:**
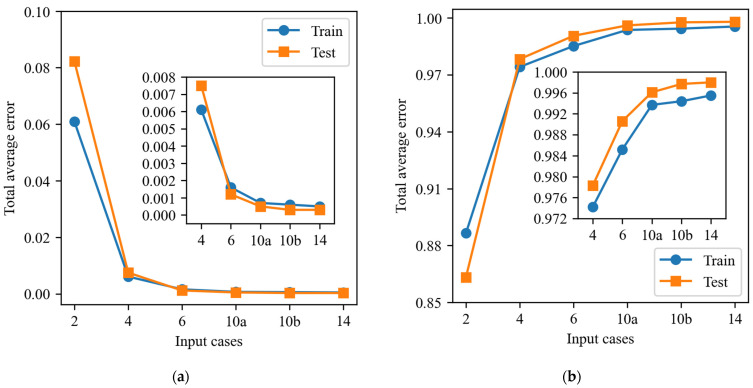
Combined evaluation metrics for FCNN models: (**a**) *MSE* mean; (**b**) *R* mean.

This study comprehensively evaluates the prediction performance of the FCNN model under six different input combinations. The average of each of *MSE* and *R* is used as the main evaluation index. These metrics offer a comprehensive view of the model’s overall performance, providing a reference point for subsequent model selection and optimization. The comprehensive evaluation metrics of the FCNN model for six combinations of inputs for the training and test sets are presented in Figure 11.

The results demonstrate that the *MSE* of the Input 2 combination is elevated, indicating that the model exhibits a considerable discrepancy in prediction. Concurrently, the correlation coefficient is diminished, indicating that the linear relationship between the prediction outcomes and the actual values is attenuated, and the overall performance is suboptimal. Input 4 exhibits a notable enhancement in performance relative to Input 2; nevertheless, a residual degree of error persists. The *R*-value indicates that it exhibits good predictive capacity, with a robust linear correlation and suitability for practical applications. The *MSE* of the Input 6 combination is further diminished, and the *R*-value approaches 1, demonstrating robust predictive capacity. The model’s fitting and generalization performance for this input combination is markedly enhanced, and it is suitable for more intricate prediction tasks.

The combination of Input 10a and Input 10b demonstrates robust performance and exhibits considerable predictive power. Specifically, Input 10a has an *MSE* of 0.0007 and an *R*-value of 0.9937 on the training set, and an *MSE* of 0.0005 and an *R*-value of 0.9961 on the test set, indicating that it is able to accurately predict the vast majority of the data points and has good generalization ability. Similarly, Input 10b has an *MSE* of 0.0006 and an *R*-value of 0.9944 on the training set, and achieves the lowest *MSE* (0.00032) and the highest *R*-value (0.9977) on the test set. The performance of these two input combinations indicates that they are highly effective at fitting and prediction, providing a valuable reference for model optimization and demonstrating potential for practical applications. The Input 14 combination exhibits the most optimal performance among all inputs, with *MSE*s of 0.0005 and 0.00025 and *R*-values as high as 0.9955 and 0.9980 for the training and test sets, respectively. It also has the lowest *MSE* and the highest *R*-value among the composite evaluation metrics of the six different input combinations, demonstrating excellent prediction accuracy and stability on both the training and test sets. Collectively, the predictive performance of the model is markedly enhanced from Input 2 to Input 14; particularly notable are the input combinations Input 10a, Input 10b, and Input 14, which demonstrate exceptional performance. These findings underscore the significance of input feature selection and the potential for substantial enhancement in the predictive capacity and generalization performance of the model through optimization of input combinations.

In the context of engineering practice, it is crucial to undertake a detailed cost analysis of the project, particularly when selecting strain gauge sensors. It is of great importance to optimize the number of sensors to be utilized in a manner that is both cost-effective and in accordance with the principle of relative minimum of prediction error [87,88]. In this study, the impact of different strain gauge configurations on the project cost is evaluated in a systematic manner, and the minimum number of strain gauge sensors to be used is determined by developing a reasonable prediction model. The objective of this analysis is to identify the optimal combination of input variables that ensures that the requirements for measurement accuracy and sensitivity are met in both laboratory and practical engineering applications. This approach enhances the reliability and validity of data while controlling the cost. This study offers theoretical support for the economic aspects of sensors and provides a crucial decision-making foundation for the practical implementation of steel tubes in related offshore wind turbine support systems.

Consequently, a comparative analysis of the comprehensive evaluation indexes of the prediction results of the test set in the FCNN model reveals that the performances of Input 10a, Input 10b, and Input 14 have reached an optimal state, with an average *MSE* below 0.001 and a linear correlation strength R of 0.99 or above. Notably, the combination of Input 10a and Input 10b demonstrates a balance between high accuracy and computational efficiency, exhibiting superior stability and generalization ability. Accordingly, the combination of Input 2, Input 4, and Input 6 has been selected for enhancement and optimization, which ensures accuracy and simultaneously provides enhanced potential for practical application. 

### 5.2. CNN Modeling for Cross-Comparison Prediction Analysis of Input Variables

**Figure 12:**
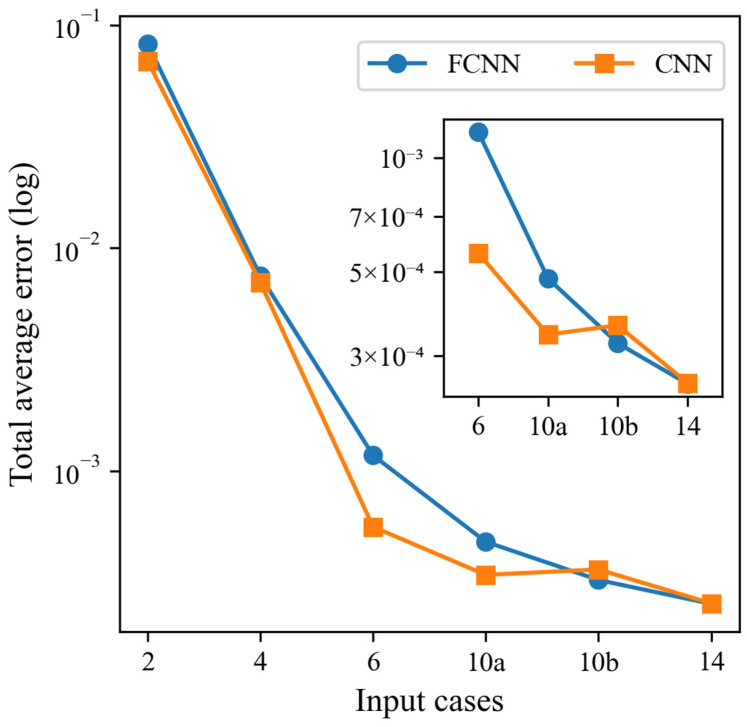
Combined evaluation metrics for FCNN model and CNN model test set-Error *MSE*.

In this study, the CNN model is employed for the modeling and analysis of Input 2, Input 4, Input 6, Input 10a, Input 10b, and Input 14. The focus should be on potential variable combinations, specifically Input 2, Input 4, and Input 6. In comparison to the FCNN model, the CNN model exhibits slightly better performance evaluation metrics for Input 2 and Input 4. Meanwhile, there is not a significant difference between Input 10a, Input 10b, and Input 14. Similarly, the CNN model demonstrates enhanced performance with an increase in the number of input variables. For Input 6, the error and correlation strength reach a level comparable to that of the evaluation metrics of the FCNN model for Input 10a, Input 10b, and Input 14. Accordingly, Input 6 of the CNN model is identified as the optimal input variable. The consolidated evaluation metrics of the FCNN and CNN test sets are presented in Table 5, and a comparative analysis of the error *MSE* is illustrated in Figure 12.

### 5.3. Interactive Coupled Mechanics Prediction System GUI for CNN Models

Based on the CNN model and input 6 identified as the optimal input variable, a law is derived for the nonlinear functional relationship between the strain of the steel tube and the reaction force. The resulting equations are presented below.

(18) $$P=w_{11}\times S1G1+w_{12}\times S1G3.5+...+w_{1n}\times S1G12+bias1$$

(19) $$Vx=w_{21}\times S1G1+w_{22}\times S1G3.5+...+w_{2n}\times S1G12+bias2$$

(20) $$Vy=w_{31}\times S1G1+w_{32}\times S1G3.5+...+w_{3n}\times S1G12+bias3$$

(21) $$Mx=w_{41}\times S1G1+w_{42}\times S1G3.5+...+w_{4n}\times S1G12+bias4$$

(22) $$My=w_{51}\times S1G1+w_{52}\times S1G3.5+...+w_{5n}\times S1G12+bias5$$

In the aforementioned equations, *w_i_*_j_ represents the weight learned by the model, which signifies the degree of influence exerted by output *i* on input *j*, and *bias* is the bias term. S1G1, S1G3.5, S1G6, S1G7, S1G9.5, and S1G12 collectively constitute the six strains of the inputs, whereas P, Vx, Vy, Mx, and My represent the five output reaction force variables.

**Figure 13:**
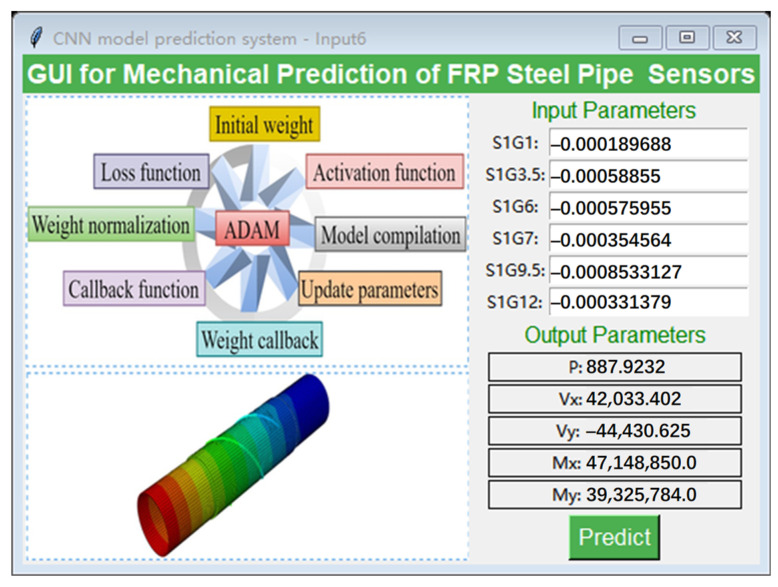
Interactive GUI for CNN model–Input 6.

In the structural design of SHM and steel tubes for offshore wind turbine support systems, the “Tkinter” library was invoked in the Python (version no.: 3.8) environment for the purposes of improving computational efficiency and the convenience of results visualization. This was achieved through the use of an intuitive parameter input and results presentation for GUI development [89,90]. The fundamental aspect of this interface is based on a trained CNN model, integrated with the principles of nonlinear functional relationships, which enables the effective processing and analysis of strain data. The user is required to input only six strain characteristic variables directly into the interface and click the “Predict” button, after which the system is able to rapidly calculate and display the prediction results for the five reaction force variables. The design of this interactive GUI, which is illustrated in Figure 13, not only streamlines the process of data input and result acquisition but also markedly enhances the user’s operational experience and calculation efficiency. This approach allows engineers to more conveniently analyze and optimize the performance of steel tubes, thereby providing timely and reliable data support for engineering design and SHM.

### 5.4. Numerical Experiments for CNN Model

To verify the proposed CNN method, the predicted values for various loads were compared with the actual load values. The load was selected as a load combination that simulates the actual situation of the offshore wind power support structure, and the load value was predicted using the six strain values measured from the numerical analysis results. The load combination was selected assuming that shear force is applied in one and two directions under a specific axial force and that the resulting bending moment is applied. At t = 0.9 s, the numerical experimental results for the two load combinations are shown in Table 6 and Table 7, and it can be seen that the actual applied loads are well predicted, and that the load prediction accuracy is high under these conditions.

## 6. Conclusions

- This study proposes an intelligent multivariate regression model based on FCNN and CNN for predicting the internal forces of FRP double-helix sensor-coupled steel tube members. The findings demonstrate that AI-driven methods have significant advantages in the field of Structural Health Monitoring (SHM). By integrating deep learning technologies with the FRP double-helix sensor system, this approach not only enhances the accuracy of internal force prediction but also makes SHM for offshore wind turbine support systems more intelligent and efficient. The results highlight the extensive application potential of AI technology in civil engineering.
- Regarding the integration of sensors and neural network models, the FRP double-helix sensor enables reliable multi-point strain data collection through optimized layout, while the CNN model optimizes variable combinations by transforming the data into image-like formats. This process ultimately determines the optimal number and placement of sensors (Input 6). This design significantly reduces the number of sensors required while maintaining prediction accuracy. The optimized approach effectively reduces project costs and resource consumption in engineering practice, validating the capability of deep learning models to address complex engineering problems.
- The practical application of this research is reflected in the development of an interactive graphical user interface (GUI) tool that enables engineers to quickly predict the internal forces of support members. This tool is suitable for real-time monitoring and rapid evaluation of offshore wind turbine support systems, providing effective support for improving the efficiency of engineering design and maintenance. The reliability of the model is verified using performance metrics such as *R*^2^, *MSE*, *MAE*, and *SMAPE*, demonstrating its advantages in addressing multi-output problems.
- Despite the significant progress achieved, certain limitations remain. The analysis in this study mainly focuses on conventional conditions within the elastic range. Future work could extend the approach to complex stress fields under nonlinear conditions. Additionally, integrating more experimental data and real-world operational tests could further validate the model’s robustness and generalizability, offering insights for designing and optimizing other support systems.
- This study presents an innovative SHM method and provides new solutions for intelligent civil engineering design. Future research will continue to explore the broader applicability and engineering scalability of this method, laying the foundation for long-term SHM and performance optimization of complex structures.

## Figures and Tables

**Table 1:** Specific physical properties of relevant materials.

Material	Diameter (mm)	Thickness (mm)	Length (mm)	Modulus of Elasticity (MPa)	Poisson’s Ratio
Steel Tube	97	3	1000	205,000	0.3
FRP Tube	100	5	300	15,000	0.2

The material properties of the double-helix wire are the same as those of the steel tubes.

**Table 2:** Strain and reaction force at the fixed end.

Variable	Number								
	1	–	50	–	500	–	800	–	1000
Time	0.001	–	0.05	–	0.5	–	0.8	–	1
S1G1	2.60643 × 10^−6^	–	1.11428 × 10^−4^	–	−7.14399 × 10^−8^	–	−7.93848 × 10^−4^	–	−1.18914 × 10^−7^
S1G2	5.12635 × 10^−7^	–	−4.98577 × 10^−6^	–	2.34286 × 10^−7^	–	1.57363 × 10^−4^	–	2.36832 × 10^−7^
S1G3	2.19770 × 10^−6^	–	9.71944 × 10^−5^	–	−2.02134 × 10^−7^	–	1.22821 × 10^−4^	–	1.67849 × 10^−7^
S1G3.5	−2.84843 × 10^−6^	–	−1.24557 × 10^−4^	–	2.89937 × 10^−7^	–	2.57832 × 10^−4^	–	−1.07993 × 10^−6^
S1G4	−1.52650 × 10^−6^	–	−7.72136 × 10^−5^	–	7.22822 × 10^−8^	–	−2.90367 × 10^−4^	–	3.43204 × 10^−9^
S1G5	−5.49184 × 10^−6^	–	−2.07579 × 10^−4^	–	1.80148 × 10^−7^	–	3.81431 × 10^−5^	–	6.26635 × 10^−7^
S1G6	6.55528 × 10^−7^	–	−1.72538 × 10^−5^	–	4.35172 × 10^−7^	–	6.73431 × 10^−4^	–	−9.15750 × 10^−7^
S1G7	−2.30069 × 10^−6^	–	−1.29847 × 10^−4^	–	−2.90252 × 10^−7^	–	5.59804 × 10^−4^	–	4.80166 × 10^−7^
S1G8	4.42004 × 10^−7^	–	−1.31939 × 10^−5^	–	6.18470 × 10^−7^	–	1.22710 × 10^−4^	–	2.02801 × 10^−7^
S1G9	4.46011 × 10^−7^	–	−1.72241 × 10^−5^	–	−3.58697 × 10^−7^	–	2.36182 × 10^−4^	–	−1.99505 × 10^−7^
S1G9.5	3.36411 × 10^−6^	–	1.91283 × 10^−4^	–	−7.28941 × 10^−7^	–	−3.13872 × 10^−5^	–	6.92708 × 10^−8^
S1G10	8.65081 × 10^−9^	–	2.85809 × 10^−6^	–	7.77037 × 10^−7^	–	3.36948 × 10^−4^	–	5.34538 × 10^−7^
S1G11	3.11608 × 10^−6^	–	1.54039 × 10^−4^	–	4.58082 × 10^−7^	–	−1.24424 × 10^−4^	–	7.79329 × 10^−7^
S1G12	3.02017 × 10^−6^	–	1.29256 × 10^−4^	–	−1.39974 × 10^−7^	–	−4.22722 × 10^−4^	–	−4.21732 × 10^−7^
P	7.00000	–	3.50000 × 10^2^	–	8.33333 × 10^2^	–	9.33333 × 10^2^	–	1.00000 × 10^3^
Vx	−3.13953 × 10^2^	–	−1.54508 × 10^4^	–	1.00398 × 10^−5^	–	4.75528 × 10^4^	–	2.15214 × 10^−5^
Vy	−6.26666 × 10^2^	–	−2.93893 × 10^4^	–	2.95524 × 10^−5^	–	2.93893 × 10^4^	–	1.17724 × 10^−5^
Mx	6.07830 × 10^5^	–	2.84635 × 10^7^	–	−6.11875 × 10^−2^	–	−2.65352 × 10^7^	–	−7.43699 × 10^−2^
My	−3.51553 × 10^5^	–	−1.72151 × 10^7^	–	−2.68673 × 10^−1^	–	4.93165 × 10^7^	–	2.69873 × 10^−2^

**Table 3:** Statistical analysis of input and output variables.

Variable	Max	Min	Average	SD	Median	Skewness	Kurtosis	Quantity
S1G1	5.18562 × 10^−4^	−1.08657 × 10^−3^	−1.34969 × 10^−4^	3.58551 × 10^−4^	−4.70620 × 10^−5^	−9.18675 × 10^−1^	7.59991 × 10^−1^	1000
S1G2	8.13798 × 10^−4^	−2.53403 × 10^−4^	9.69276 × 10^−5^	2.31278 × 10^−4^	3.83514 × 10^−5^	1.41300	2.15216	1000
S1G3	4.34290 × 10^−4^	−7.02724 × 10^−4^	1.29007 × 10^−5^	2.86414 × 10^−4^	5.22401 × 10^−5^	−8.24923 × 10^−1^	1.45556 × 10^−1^	1000
S1G3.5	4.74011 × 10^−4^	−9.89815 × 10^−4^	−3.98808 × 10^−5^	3.29115 × 10^−4^	−2.64781 × 10^−5^	−8.52608 × 10^−1^	7.05133 × 10^−1^	1000
S1G4	6.15423 × 10^−4^	−4.35879 × 10^−4^	3.49296 × 10^−5^	2.67868 × 10^−4^	3.02154 × 10^−6^	4.22521 × 10^−1^	−3.96468 × 10^−1^	1000
S1G5	6.39601 × 10^−4^	−7.68633 × 10^−4^	6.71803 × 10^−5^	3.57381 × 10^−4^	5.35384 × 10^−5^	−5.42648 × 10^−1^	−1.73634 × 10^−1^	1000
S1G6	6.76991 × 10^−4^	−6.05781 × 10^−4^	−4.74632 × 10^−5^	2.68679 × 10^−4^	−1.00409 × 10^−5^	−6.03959 × 10^−2^	7.53766 × 10^−1^	1000
S1G7	5.63200 × 10^−4^	−9.36714 × 10^−4^	−1.07080 × 10^−4^	3.31270 × 10^−4^	−6.59848 × 10^−5^	−6.01145 × 10^−1^	4.84777 × 10^−1^	1000
S1G8	8.09240 × 10^−4^	−3.33270 × 10^−4^	9.73150 × 10^−5^	2.38915 × 10^−4^	4.02714 × 10^−5^	1.00508	1.40003	1000
S1G9	4.04137 × 10^−4^	−8.16492 × 10^−4^	8.43682 × 10^−6^	3.02262 × 10^−4^	5.81366 × 10^−5^	−1.19401	7.13884 × 10^−1^	1000
S1G9.5	5.59487 × 10^−4^	−1.02465 × 10^−3^	−2.58145 × 10^−5^	3.40170 × 10^−4^	−3.33002 × 10^−5^	−5.91806 × 10^−1^	6.22294 × 10^−1^	1000
S1G10	5.15147 × 10^−4^	−4.84967 × 10^−4^	6.29947 × 10^−5^	2.39223 × 10^−4^	5.98854 × 10^−5^	−3.82613 × 10^−1^	−1.37343 × 10^−1^	1000
S1G11	6.92355 × 10^−4^	−5.15911 × 10^−4^	4.62883 × 10^−5^	2.74684 × 10^−4^	1.60620 × 10^−6^	2.81283 × 10^−1^	−1.18549 × 10^−1^	1000
S1G12	8.29720 × 10^−4^	−5.32430 × 10^−4^	−1.06863 × 10^−5^	2.81636 × 10^−4^	6.73036 × 10^−6^	7.96226 × 10^−1^	8.34247 × 10^−1^	1000
P	1000	0	7.99699 × 10^2^	1.83504 × 10^2^	8.33333 × 10^2^	−2.22521	5.64135	1000
Vx	50,000	−50,000	3.15312 × 10^−8^	3.53438 × 10^4^	1.00398 × 10^−5^	−2.68136 × 10^−12^	−1.49999	1000
Vy	4.99013 × 10^4^	−4.99013 × 10^4^	7.99676 × 10^−5^	3.53093 × 10^4^	3.39514 × 10^−6^	−2.26788 × 10^−9^	−1.49999	1000
Mx	52,078,600	−52,077,100	−2.86954 × 10^2^	3.53699 × 10^7^	−6.11875 × 10^−2^	4.06369 × 10^−6^	−1.48935	1000
My	50,355,900	−50,354,200	6.97984 × 10^2^	3.54052 × 10^7^	0	1.48570 × 10^−5^	−1.48917	1000

SD = Standard Deviation.

**Table 4:** Advantages and disadvantages of FCNN model and CNN model.

**Model**	**Advantages**	**Disadvantages**
FCNN	The model is capable of processing a multitude of input data formats, both linear and nonlinear, and is well suited for a diverse array of tasks, including regression and classification. The generality of this approach renders the structure relatively simple and facilitates comprehension and implementation.	Each neuron is connected to all neurons in the preceding layer. The number of parameters increases exponentially with the number of layers. The computational cost is high, and overfitting is a significant concern. The spatial information present in the input data, such as images, cannot be effectively utilized, resulting in the loss of spatial information. Each pixel point is treated as an independent feature, and there is no mechanism in place to capture local features.
CNN	The convolutional layer and pooling layer facilitate the effective extraction of local and global spatial features in the input data, thereby enhancing the model’s generalization ability and preventing overfitting. The convolution operation and the movement of the convolution kernel effectively reduce the number of parameters through the parameter sharing mechanism, thereby reducing the computational burden and conserving memory and processing time.	The structure is more intricate and necessitates a more comprehensive hyper-parameter tuning and design process. The model is not sufficiently flexible to accommodate non-image data. However, it is possible to transform non-image data into suitable inputs through the process of feature engineering.

**Table 5:** Combined evaluation metrics for FCNN model and CNN model test set.

Input	FCNN					CNN				
	R^2^	MSE	MAE	SMAPE	R	R^2^	MSE	MAE	SMAPE	R
Input 2	0.746	0.082	0.175	1.124	0.863	0.768	0.069	0.181	1.113	0.877
Input 4	0.954	0.007	0.054	1.100	0.978	0.967	0.007	0.049	1.104	0.984
Input 6	0.980	0.0012	0.023	1.102	0.991	0.993	0.00056	0.018	1.103	0.997
Input 10a	0.992	0.0005	0.016	1.101	0.996	0.993	0.00034	0.013	1.105	0.997
Input 2	0.746	0.082	0.175	1.124	0.863	0.768	0.069	0.181	1.113	0.877
Input 10b	0.993	0.00032	0.011	1.103	0.998	0.992	0.00036	0.011	1.101	0.996
Input 14	0.996	0.00025	0.011	1.101	0.998	0.995	0.00025	0.011	1.102	0.998

**Table 6:** Combined evaluation metrics for CNN model test set 1.

Microstrain		Forces [A]		Predicted Forces [B]	Error [A−B]
S1G1	−15.8499	P [kN] Vx [kN] Vy [kN] Mx [kN·m] My [kN·m]	0.966667 0.117557 −1.771 × 10^−15^ −6.47132 × 10^−8^ 2.02018	0.982713 0.122016 −3.2504 × 10^−15^ −6.30282 × 10^−8^ 1.96190	−0.016046 −0.004459 1.4794 × 10^−15^ −0.168498 × 10^−8^ 0.05828
S1G3.5	−2.75776				
S1G6	−6.70106				
S1G7	19.8599				
S1G9.5	7.70355				
S1G12	0.186638				

**Table 7:** Combined evaluation metrics for CNN model test set 2.

Microstrain		Forces [A]		Predicted Forces [B]	Error [A−B]
S1G1	−7.92872	P [kN] Vx [kN] Vy [kN] Mx [kN·m] My [kN·m]	1.93333 0.235114 0.190211 −0.425514 0.996432	1.83586 0.229871 0.189499 −0.419330 0.988110	0.0974706 0.0052433 0.00071216 −0.0061838 0.0083224
S1G3.5	1.83893				
S1G6	−3.14737				
S1G7	8.09374				
S1G9.5	2.26935				
S1G12	−1.50729				

## Data Availability

The original contributions presented in this study are included in the article. Further inquiries can be directed to the corresponding author.
